# A comparison between young and old patients with triple-negative breast cancer: biology, survival and metastatic patterns

**DOI:** 10.1007/s10549-020-05727-x

**Published:** 2020-06-10

**Authors:** Anna-Karin Tzikas, Szilard Nemes, Barbro K. Linderholm

**Affiliations:** 1 Department of Oncology, Institute of Clinical Sciences, Sahlgrenska Academy at University of Gothenburg, Gothenburg, Sweden; 2 Department of Oncology, NU Hospital Group, Uddevalla, Sweden; 3 Department of Orthopaedics, Institute of Clinical Sciences, Sahlgrenska Academy at University of Gothenburg, Gothenburg, Sweden; 4 Department of Oncology, Sahlgrenska University Hospital, Gothenburg, Sweden

**Keywords:** Triple negative breast cancer, Chemotherapy, Biology, Elderly, Age, Survival

## Abstract

**Purpose:**

To determine the biology, recurrence rate, metastatic patterns and survival times in primary triple-negative breast cancer (TNBC) with focus on the comparison between younger and elderly patients.

**Methods:**

Patients with primary TNBC stage I–IV diagnosed from 2007 to 2015 were identified and information on tumor biology, stage, treatment, recurrences and death recorded.

**Results:**

A total of 524 patients, median age 60 years (range 24–94) with a median follow-up of 55 months (range 0–129) were identified. Stage was similar in younger (< 40 years) (*n* = 58) and older (> 74 years) (*n* = 96) patients (*p* = 0.37). A statistically significant difference was found concerning histopathologic grade (*p* = 0.006) and Ki67 (median 80% versus 70%; *p* = 0.002) but not for LVI (*p* = 0.9) with more aggressive tumors among younger patients. Adjuvant/neoadjuvant chemotherapy was more frequently given to younger compared with older patients (96% versus 12%; *p* = 0.0005). Only brain (*p* = 0.016) and liver (*p* = 0.047) metastases were more often registered among younger patients while other locations were similar. Shorter survival times, recurrence-free survival (RFS), distant disease-free survival (DDFS) and breast cancer-specific survival (BCSS) were found in the older group, although not after adjusting for adjuvant/neoadjuvant chemotherapy. Most deaths (68%) in the older group were caused by TNBC. When comparing patients > 75 years (*n* = 92) with ≤ 75 years (*n* = 432), a worse outcome among older was also observed: RFS (*p* = 0.00012), DDFS (*p* = 0.00041), BCSS (*p* < 0.0001) and survival following distant metastasis (*p* = 0.0064)

**Conclusions:**

Primary TNBC in younger patients is more often of poor differentiation grade and highly proliferative compared with older patients. The majority of older patients still have grade III tumors with a Ki67 > 60% and outcome is poor. Few older patients in our study were treated with chemotherapy both in adjuvant and palliative setting, underlining the need for more prospective trials and treatment options suitable for this patient population.

## Introduction

Much research has focused on triple-negative breast cancer (TNBC) in young breast cancer patients. TNBC constitutes a larger proportion among younger than older breast cancer patients [1, 2]. With an aging population and increasing breast cancer incidence with age, a significant number of TNBC cases arise in elderly. Older patients with breast cancer are underrepresented in randomized clinical trials [3]. Few studies have compared the prognosis of younger and older women with TNBC [4].

Breast cancer (BC) is the most common cancer in women worldwide with more than 2 million new cases and 627 000 deaths in 2018 [5]. TNBC accounts for about 15% [6] of breast cancer cases but is the cause of a disproportional number of breast cancer deaths. TNBC lacks the expression of the estrogen receptor (ER), progesterone receptor (PR) and human epidermal growth factor receptor 2 (HER2) [7]. TNBC tends to behave more aggressively and remains a poorly defined and heterogeneous subset of breast cancer with an adverse prognosis [7]. The risk of distant recurrence typically peaks between 1 and 3 years after diagnosis and declines rapidly thereafter [8]. Unlike ER-positive and HER2-positive breast cancer, there are no approved targeted adjuvant treatments available and chemotherapy remains the sole medical intervention with a proven effect on long-term survival [6]. There is a larger benefit to adjuvant/neoadjuvant chemotherapy in ER-negative compared with ER-positive breast cancer [9]. Up to 20% of TNBC cases harbor a BRCA mutation, particularly in BRCA1 and the risk of having a BRCA mutation is higher among young TNBC patients [10].

Is TNBC the same disease in older as in younger patients? We conducted this study to determine the biology, recurrence rate, metastatic patterns and survival from diagnosis of primary breast cancer, as well as from diagnose of distant metastasis in a population-based Swedish cohort of women with primary TNBC, with focus on the comparison between younger and older patients.

## Material and methods

### Patients

Patients with primary TNBC stage I–IV from 2007 through 2015 were identified through the Swedish regional breast cancer registry. The completeness of the regional breast cancer registry regarding ER, PR and HER2 during these years exceeds 96% [11]. TNBC was defined as ER negative, PR negative (≤ 10% expression of ER and PR) and HER2 negative (either 0 to 1+ by IHC or IHC 2+ and FISH/SISH negative) according to Swedish national guidelines [12]. Immunostaining and scoring of ER, PR, HER2 and Ki67 were routinely evaluated at the time of diagnosis of the primary tumor at the department of pathology, Sahlgrenska University Hospital and Södra Älvsborg Hospital. The data used in this study were obtained from the pathology report. Clinical data were extracted from patients’ charts. For comparison between younger and older patients, we defined younger as < 40 years and older as > 74 years at diagnosis of primary TNBC as these patients do not participate in the Swedish mammography screening program. In a separate analysis, we divided all patients in ages < 40, 41–50, 51–65, 66–75 and > 75 years.

Patients were excluded if the cancer was found to be of other origin than breast cancer, if the breast cancer was ER, PR or Her 2 positive or if the cancer was found to be a recurrence of an earlier diagnosed cancer. For the validation of the pathology report, we excluded cases not operated in the region or diagnosed other years than selected.

Primary tumor stage was coded using the AJCC TNM staging system, 8th edition [13]. Pathologic report was used for TNM classification if the patient had primary surgery. If the patient did not have primary surgery, as when treated with neoadjuvant chemotherapy, the largest measurement with mammography or ultrasound was used. When a patient was diagnosed with bilateral TNBC, data from the most advanced tumor were chosen for the analysis. If the patient had primary TNBC in the axilla, *T* status was stated as *T*X. If no axillary surgery was performed, *N* status was stated as *N*0 if there were no clinical or radiological signs of lymph node involvement. The diagnosis of *M*1 disease relies on pathological examination and/or radiology (CT, MRI) together with clinical examination. If metastases were identified within 3 months of diagnosis, this was stated as de novo stage IV disease. For comparison, histology was divided into ductal, lobular, medullary, apocrine, metaplastic and other carcinoma.

### Treatment and follow-up

The type of breast and axillary surgery was registered along with adjuvant/neoadjuvant chemotherapy and radiation therapy. Adjuvant/neoadjuvant chemotherapy was divided into the following: *A*—anthracycline based (FEC or EC × 6); *A* + *T*—anthracycline and taxane based (EC or FEC × 3 plus docetaxel × 3 or Paclitaxel × 9–12, in some cases combined with carboplatin); *T*—taxane based (Docetaxel × 6 or Paclitaxel × 9–12) or *CMF*—CMF based (CMF × 6 or CMF × 3 + EC or FEC × 3 or CMF + Paclitaxel × 9–12).

The planned follow-up time was 5 years from diagnosis of primary TNBC. The recurrence and survival status was obtained through medical record review. The patients were considered to be recurrence free if still living in the region at last day of follow-up and with no signs or symptoms of recurrence. The diagnosis of recurrence relies on pathological examination and/or radiology (CT, MRI, ultrasound, mammography) together with clinical examination. Date of recurrence was defined as first date of pathological, radiological of clinical proof or recurrence. Cause of death was extracted from patients’ charts. Death was defined as breast cancer related when breast cancer was listed as a contributing cause of death and the patient had metastatic TNBC disease. Data on metastatic sites as well as administration of radiotherapy and number of lines of palliative chemotherapy were also registered. If a patient had moved to another region, she was referred to as lost to follow-up.

### Statistical analysis

Recurrence-free survival was defined as survival from date of diagnosis until date of first recurrence, loco regional or systemic. Distant disease-free interval was calculated as time from diagnosis to distant recurrence. Breast cancer-specific survival was calculated from the date of diagnosis to date of death due to breast cancer. Patients with de novo metastatic disease were excluded from the calculations of recurrence-free survival, distant disease-free survival and breast cancer-specific survival. Survival following distant metastasis was calculated from the date of distant recurrence or diagnosis of metastatic disease to the date of death from any cause or the date of last follow-up.

We used the *χ*^2^-test to test association between categorical variables, and the t test for comparison of continuous variables. Survival curves estimated by the Kaplan Meier estimator were compared by the log-rank test. Cox proportional hazards models were used to estimate the univariate and multivariate hazard ratios (HRs) and associated 95% confidence intervals. We used the models to examine the impact of age groups on recurrence-free survival, distant disease-free survival, breast cancer-specific survival and survival following distant metastasis. Analyses were adjusted for tumor size, lymph node involvement and grade and in a separate analysis we also adjusted for chemotherapy. We tested the assumption of proportionality with graphical examination and significance testing and no deviations were observed (smallest p value = 0.16). In order to depict the effect of age on survival, we constructed a model per outcome where all 524 subjects were kept and age at diagnosis was modeled as a continuous variable with the help of penalized splines with 4 knots. To facilitate visualization, the reference was set to the average age of all subjects, 60 years.

## Results

### Patients

**Fig. 1:**
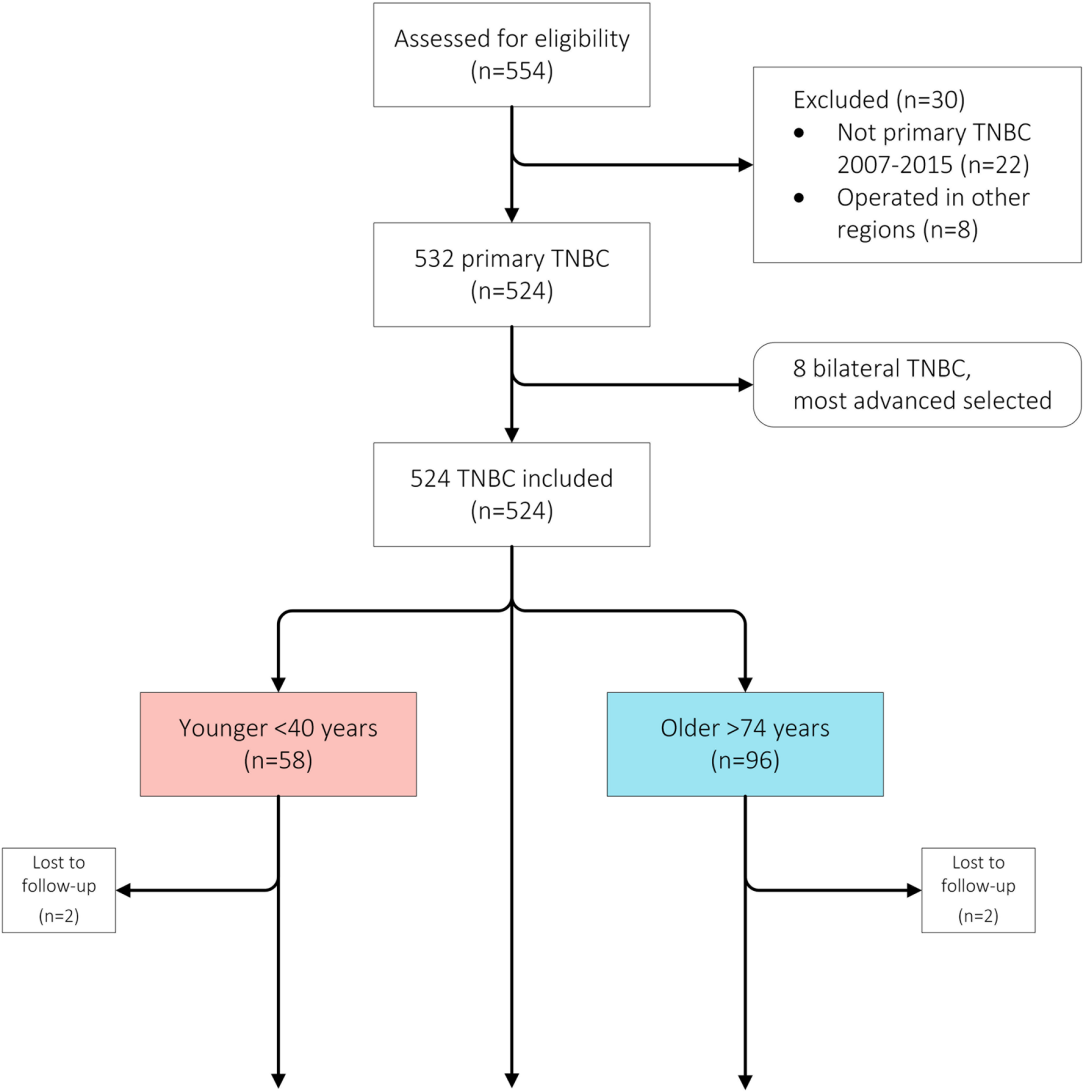
Consolidated Standards Of Reporting Trials (CONSORT) diagram showing the selection of patients and number of patients excluded

A total of 524 patients, all women, were identified including 58 patients under 40 years and 96 patients above 74 years when diagnosed with TNBC. The selection of patients is summarized in Fig. 1. Baseline characteristics and adjuvant treatments are displayed in Table 1. Median age at diagnosis was 60 years (range 24–94). The stages at diagnosis were as follows: stage I (26%); stage II (49%); stage III (22%) and stage IV (3%). Most tumors (87.4%) were ductal invasive carcinomas of poor histopathological grade (80% grade 3). Histology and clinical stage at diagnosis did not differ between age groups. Only 5 out of 524 tumors were 1–10% ER or PR positive. A statistically significant difference was found concerning histopathologic grade (*p* = 0.006) and Ki67 (median 80% versus 70%; *p* = 0.002) but not for LVI (*p* = 0.9) with a higher proportion of poorly differentiated high proliferative tumors among younger patients. Older patients were more likely to have mastectomy (*p* = 0.0004), less axillary surgery (*p* = 0.0004) and less adjuvant/neoadjuvant chemotherapy (*p* = 0.0005). In the whole cohort, 395 out of 506 patients stage I–III (78%) received adjuvant/neoadjuvant chemotherapy. Adjuvant/neoadjuvant chemotherapy was only delivered to 11 out of 89 older stage I–III patients (12%) compared with 55 out of 57 (96%) in the younger group. Type of chemotherapy treatment also differed between the age groups with more anthracycline and taxane based treatments in the younger group (*p* = 0.004). Older patients received less adjuvant radiation therapy (*p* = 0.0004).

**Table 1:** Baseline characteristics of 524 patients with diagnose of triple-negative breast cancer

	All patients *n* = 524 *N* (%)	Younger < 40 years *n* = 58 *N* (%)	Older > 74 years *n* = 96 *N* (%)	Difference younger/older *p* value
Age, median years (range)	60 (24–94)	36 (24–39)	81 (75–94)	
Histology				0.26
Ductal	458 (87.4)	55 (94.8)	85 (88.5)	
Lobular	12 (2.3)	1 (1.7)	2 (2.1)	
Medullary	14 (2.7)	1 (1.7)	0	
Apocrine	10 (1.9)	0	2 (2.1)	
Metaplastic	15 (2.9)	0	5 (5.2)	
Other	15 (2.9)	1 (1.7)	2 (2.1)	
Primary tumor, *T*	(470p/54c)	(48p/10c)	(89p/7c)	0.91
*T*X	7 (1.3)	1 (1.7)	1 (1.0)	
*T*1	170 (32.4)	14 (24.1)	18 (18.8)	
*T*2	253 (48.3)	30 (51.7)	50 (52.1)	
*T*3	69 (13.2)	12 (20.7)	20 (20.8)	
*T*4	25 (4.8)	1 (1.7)	7 (7.3)	
Regional lymph nodes, *N*	(450p/74c)	(48p/10c)	(71p/25c)	0.35
*N*0	316 (60.3)	37 (63.8)	55 (57.3)	
*N*1	126 (24.0)	15 (25.9)	21 (21.9)	
*N*2	45 (8.6)	2 (3.4)	10 (10.4)	
*N*3	37 (7.1)	4 (6.9)	10 (10.4)	
Distant metastasis, *M*				0.22
*M*0	506 (96.6)	57 (98.3)	89 (92.3)	
*M*1^a^	18 (3.4)	1 (1.7)	7 (7.3)	
Stage				0.37
I	134 (25.6)	9 (15.5)	12 (12.5)	
II	256 (48.9)	37 (63.8)	51 (53.1)	
III	115 (21.9)	11 (19.0)	26 (27.1)	
IV	18 (3.4)	1 (1.7)	7 (7.3)	
LVI				0.9
Present	127 (24.2)	15 (25.9)	22 (22.9)	
Not present	334 (63.7)	36 (62.1)	63 (65.6)	
Missing	63 (12.0)	7 (12.1)	11 (11.5)	
Grade				0.006
Grade 1	9 (1.7)	1 (1.7)	0 (0)	
Grade 2	96 (18.3)	2 (3.4)	18 (18.8)	
Grade 3	409 (78.1)	53 (91.4)	77 (80.2)	
Missing	10 (1.9)	2 (3.4)	1 (1.0)	
Ki 67, median (range)	70 (1–100)	80 (20–95)	70 (1–95)	0.002
Breast surgery				0.0004
BCS	208 (39.7)	21 (36.2)	11 (11.5)	
Mastectomy^b^	302 (57.6)	35 (60.3)	82 (85.4)	
Others^c^	14 (2.7)	2 (3.4)	3 (3.1)	
Axillary surgery				0.0004
Sn	266 (50.8)	29 (50.0)	29 (30.2)	
Sn + ALND	69 (13.2)	10 (17.2)	8 (8.3)	
ALND	157 (30.0)	18 (31.0)	37 (38.5)	
No axillary surgery	32 (6.1)	1 (1.7)	22 (22.9)	
Adj/neoadj chemotherapy				0.0005
Adjuvant	380 (72.5)	53 (91.4)	9 (9.4)	
Neoadjuvant	15 (2.9)	2 (3.4)	2 (2.1)	
No	111 (21.2)	2 (3.4)	78 (81.3)	
No due to stage IV	18 (3.4)	1 (1.7)	7 (7.3)	
Type of adj/neoadj chemotherapy				0.004
A based	151 (28.9)	16 (27.6)	6 (6.3)	
*A* + *T* based	220 (42.0)	38 (67.2)	1 (1.0)	
*T* based	14 (2.7)	1 (1.7)	2 (2.1)	
CMF based	10 (1.9)	0 (0)	2 (2.1)	
Adjuvant radiotherapy				0.0004
Yes	336 (64.1)	40 (69.0)	25 (26.0)	
No	168 (32.1)	17 (29.3)	63 (65.6)	
Not provided due to stage IV	20 (3.8)	1 (1.7)	8 (8.3)	

Comparisons are made between the younger (*n* = 58) and older (*n* = 96) patients

*p/c* number of patients that were pathologically assessed/clinically assessed, *BCS* breast-conserving surgery, *A* anthracycline, *T* taxane, *CMF* cyclophosphamide, methotrexate and 5 fluorouracil, *ALND* axillary lymph node dissection

^a^Distal metastases diagnosed within 3 months of diagnosis

^b^Including BCS followed by mastectomy

^c^Including core biopsy, extirpation

### Follow-up and survival

Patients had a median follow-up duration of 55 months (range 0–129). Ten patients were lost to follow-up due to relocation including 2 from the younger group and 2 from the elderly group. Out of 524 patients, 372 (71%) were free from recurrences and 11 patients were treated for local recurrence without developing metastatic disease during follow-up. A total of 141 patients (27%) were diagnosed with distant metastases; 123 developed distant metastases during follow-up and 18 patients had primary stage IV disease. 170 patients died during follow-up; 134 patients (79%) died due to breast cancer and 36 due to other causes. In the younger group (*n* = 58), one patient was diagnosed with primary stage IV disease and 14 developed a distant recurrence. Thirteen patients in the younger group died during follow-up, all due to breast cancer. In the older group (*n* = 96), 7 patients had primary stage IV disease and 35 developed a distant recurrence during follow-up. Fifty seven of the older patients died during follow-up, 39 (68%) due to breast cancer and 18 (32%) due to other causes.

**Fig. 2:**
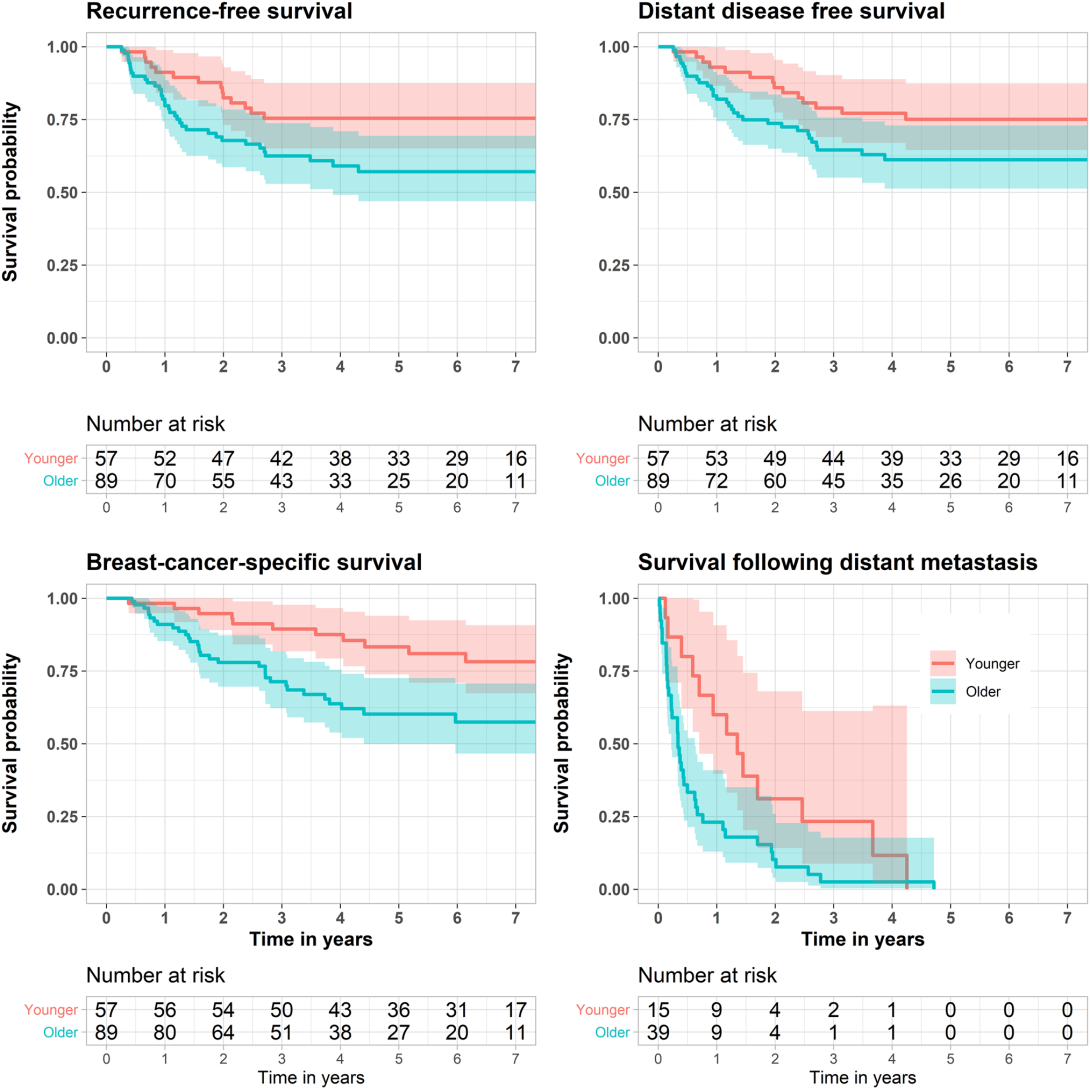
Kaplan–Meier plots depicting survival according to age group—younger *versus* older patients: recurrence-free survival, distant disease-free survival, breast cancer-specific survival and survival following distant metastasis

**Fig. 3:**
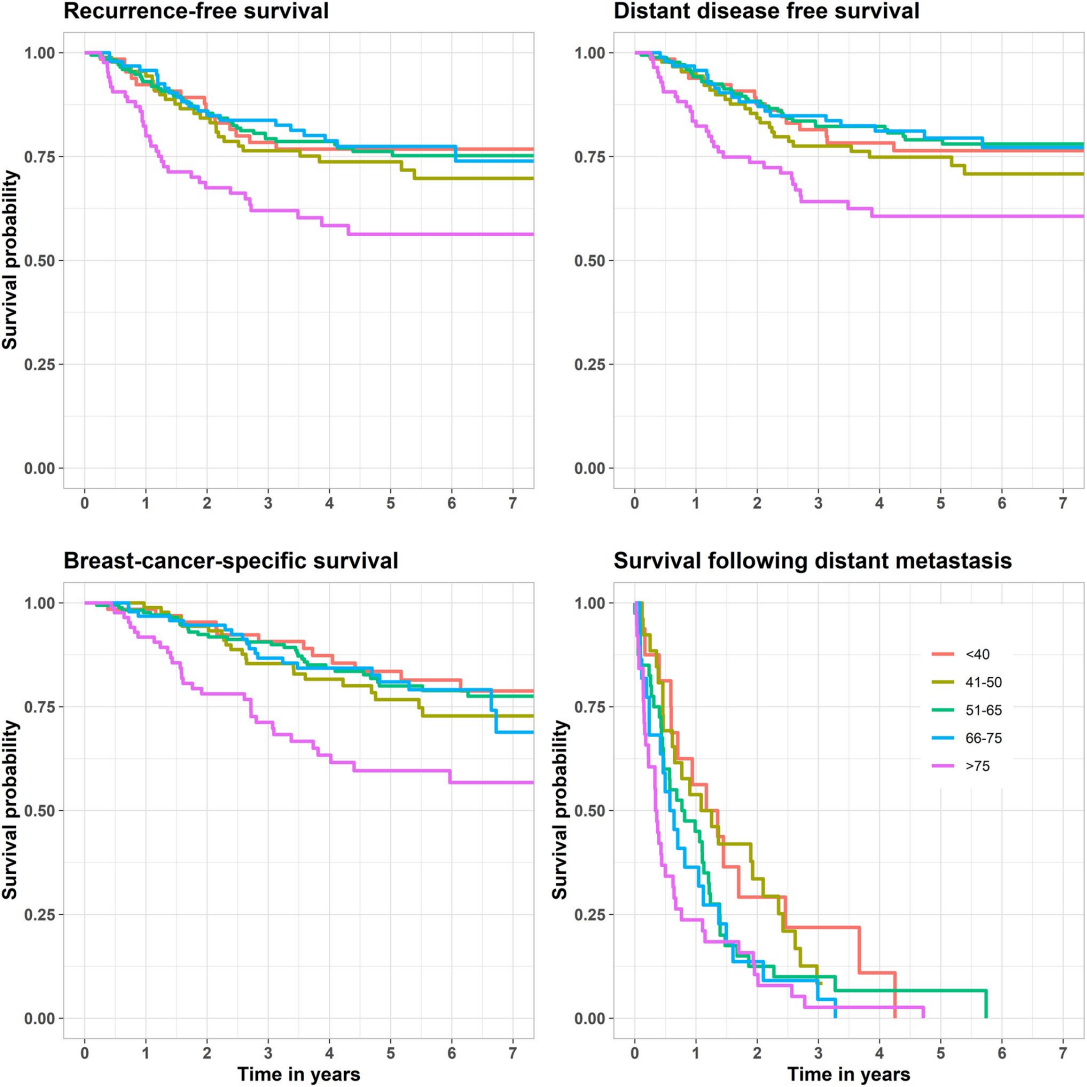
Kaplan–Meier plots depicting survival according to age group < 40, 41–50, 51–65, 66–75 and > 75 years: recurrence-free survival, distant disease-free survival, breast cancer-specific survival and survival following distant metastasis

**Fig. 4:**
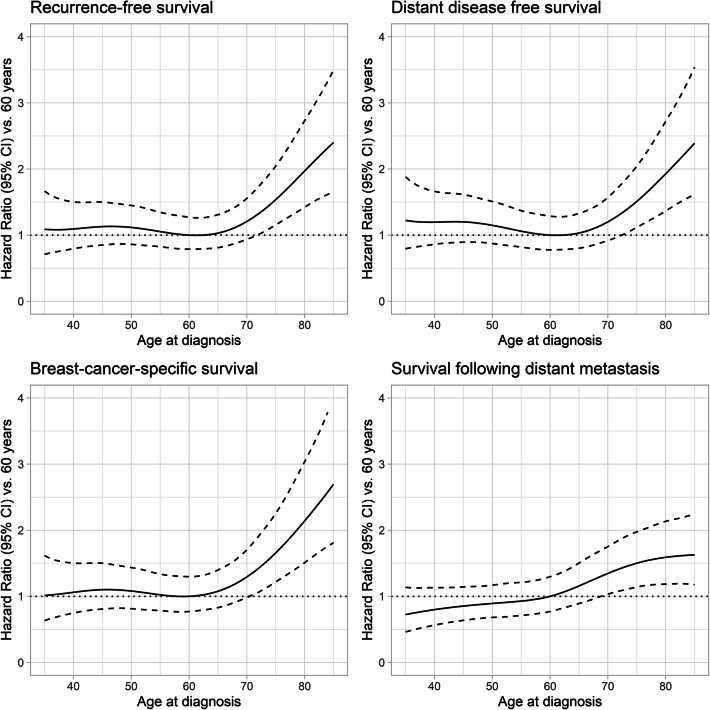
Effect of age on survival probabilities

Recurrence-free survival, distant disease-free survival, breast cancer-specific survival and survival following distant recurrence are shown in Fig. 2. Unadjusted and adjusted HR and 95% CI are shown in Table 2. Shorter survival times were found among older patients also after adjusting for tumor size, lymph node involvement and grade: recurrence-free survival (HR 2.22; 95% CI 1.12–4.43), distant disease-free survival (HR 2.13; 95% CI 1.07–4.27) and breast cancer-specific survival (HR 2.78; 95% CI 1.33–5.81) but this was not significant for survival following distant metastases (HR 1.36; 95% CI 0.62–2.99). When adjusting also for adjuvant/neoadjuvant chemotherapy, the difference in survival between the two groups disappeared. Survival in all age groups is shown in Fig. 3. The survival curves deviated between patients under and over 75 years. Worse survival was found in patients > 75 (*n* = 92) compared with patients ≤ 75 years (*n* = 432): recurrence-free survival (*p* = 0.00012), distant disease-free survival (*p* = 0.00041), breast cancer-specific survival (*p* < 0.0001) and survival following distant metastasis (*p* = 0.0064). The effect of age on survival is shown in Fig. 4.

**Table 2:** Age group-specific survival and excess hazard in the older group

Endpoint	Unadjusted		Adjusted^a^		Adjusted^b^	
	HR	95% CI	HR	95% CI	HR	95% CI
Recurrence-free survival	1.95	1.05; 3.63	2.22	1.12; 4.43	0.69	0.17; 2.80
Distant disease-free survival	1.78	0.95; 3.35	2.13	1.07; 4.27	0.58	0.15; 2.30
Breast cancer-specific survival	2.36	1.21; 4.61	2.78	1.33; 5.81	0.74	0.17; 3.18
Survival following distant metastasis	1.57	0.73; 2.99	1.36	0.62;2.99	0.51	0.12; 2.10

^a^Analyses were adjusted for tumor size, lymph node involvement and grade

^b^Analyses were adjusted for tumor size, lymph node involvement, grade and chemotherapy (adjuvant/neoadjuvant chemotherapy in breast cancer-specific, recurrence-free and distant disease-free survival and palliative chemotherapy in survival following distant metastasis)

### Treatment and outcome after distant metastasis

Treatments in patients diagnosed with distant metastasis are shown in Table 3. Median distant disease-free interval among all patients who developed metastatic disease was 15 months: 24 among younger and 13 among older patients, although the difference between age groups did not reach statistical significance (*p* = 0.06). Fifty six % in the whole group received palliative chemotherapy. Few patients received more than 1 line of palliative chemotherapy. Only 10% in the older group received palliative chemotherapy compared with 93% in the younger group (*p* = 0.0004). Fifty five % in the whole group received palliative radiotherapy and there was no significant difference between age groups (*p* = 0.07).

**Table 3:** Treatments in patients diagnosed with distant metastasis

	All patients *n* = 141	Younger < 40 years *n* = 15	Older > 74 years *n* = 39	Difference younger/older *p* value
Distant disease-free interval (months) median, CI	14.8 (11.3; 23.8)	23.6 (13.7; 37.7)	13.0 (10.1; 22.5)	0.064
Palliative chemotherapy, *N* (%)				0.0004
Yes	79 (56.0)	14 (93.3)	4 (10.3)	
No	62 (44.0)	1 (6.7)	35 (89.8)	
Lines of chemo, median (range)	1 (0–6)	3 (0–4)	0 (0–4)	NA
Palliative radiotherapy, *N* (%)				0.07
Yes	77 (54.6)	9 (60)	15 (38.5)	
No	64 (45.4)	6 (40)	24 (61.5)	

### Localization of distant metastasis

**Fig. 5:**
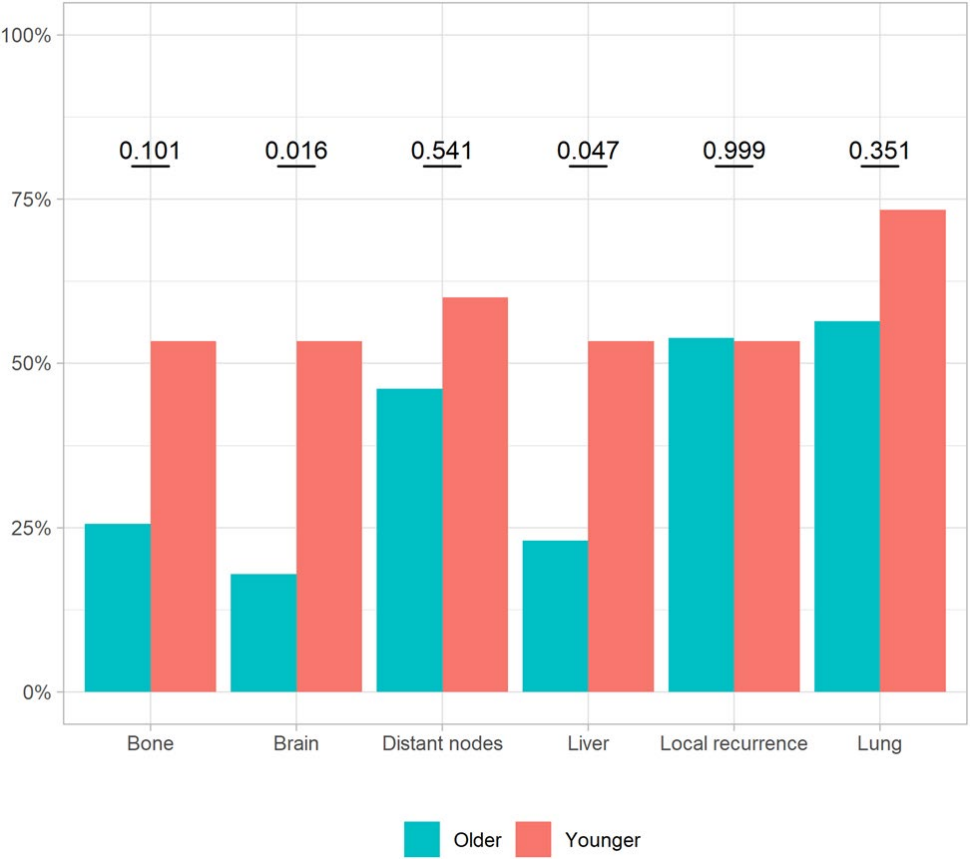
Proportion of patients with metastasis in different organs according to age group—younger *versus* older patients

In the whole cohort, 60% out of 141 patients with stage IV disease developed distant metastases to the lung, 45% to distant nodes, 43% to bone/bone marrow, 40% to liver, 40% to brain and 39% also had local recurrence. Patterns of recurrence were similar in the young and the old group concerning lung (*p* = 0.351), distant nodes (*p* = 0.541), bone (*p* = 0.101) and local recurrence (*p* = 0.999). Brain and liver metastases were more often registered among younger patients compared with older (*p* = 0.016) (*p* = 0.047), respectively (Fig. 5).

## Discussion

Previous data have shown improved survival in women with BC over time but this is not true to the same extent in patients > 75 years old [14]. The major findings of this investigation were that recurrence-free, distant disease-free and breast cancer-specific survival was statistically significantly worse in older patients with TNBC diagnosed at > 74 years compared to the younger group. To avoid selection bias with a more advanced tumor stage among elderly patients, the cut-off levels for the separation of younger and elderly were prospectively set to 40 and 74 years, respectively, as this is the age limits for the national mammogram screening program. In addition, when patients were split into five age groups, survival following treatment for primary TNBC was markedly worse for the older group, while the other four had a similar outcome.

We could not demonstrate that older patients had diagnose of BC at a more advanced stage. However, a larger proportion of 26% among the elderly were solely assessed clinically in the axilla. This is higher compared with a large registry based publication [15]. Although our patients are slightly older, only 12% of patients above 70 years were left without axillary surgery in their study. Thus, lymph node involvement among older might be underdiagnosed.

The incidence of TNBC is higher among younger patients. As BC is a much more common disease among older patients, there is still a substantial number of old patients with this diagnose. We show that the median age at diagnosis in this population-based cohort of TNBC was 60 years, which is close to the median age at diagnosis for all BC. A minor proportion in the older group of around 10% received chemotherapy, both in the adjuvant and palliative setting. In addition, postoperative radiotherapy was delivered to fewer older patients, but this is likely due to more mastectomies in this group and not to actively avoiding treatment. So far, chemotherapy is the sole systemic treatment with proved long-term efficacy for patients with TNBC, although newer compounds have been introduced in the palliative setting [16–18]. Data from clinical trials where chemotherapy has been examined in the older population are few. Previous published studies have shown excess mortality among elderly BC patients and that chemotherapy is less commonly used than in younger individuals [18]. However, two large studies where patients in the US Surveillance, Epidemiology and End Results Program (SEER) database were investigated specifically addressed the use and effect of adjuvant chemotherapy in older patients, defined as > 65 years. The results concordantly showed a reduction in mortality by adjuvant chemotherapy in patients with hormone receptor-negative node-positive BC [19, 20], with consistent results in the subgroup of patients’ aged 70 years or older [18]. Patients studied received adjuvant chemotherapy during the 1990s, and both publications demonstrate an increase in chemotherapy use during the study periods [17, 18]. It is not possible to extract the TNBC patients’ as HER2 status not was determined during this period. With regard to type of chemotherapy, CMF-based regimens were the most common during the initial years, but this regimen was gradually replaced by anthracycline-containing regimens which also included taxanes for a proportion of patients [18]. Probably, these patients received more moderate adjuvant chemotherapy than todays’ recommendations, but still resulted in a patient gain.

A recently published large registry study from two regions in England determined the use of and effect of chemotherapy in > 10,000 patients above the age of 70 years from 2002 to 2012 [21]. Among patients aged 70–79 years, the study identified an increase in the use of chemotherapy from 2002 as well as a reduction in breast cancer mortality. Moreover, a prospective trial demonstrated that standard AC or CMF is better than Capecitabine in the adjuvant setting for elderly patients [22], while no advantage could be documented for anthracycline-containing chemotherapy compared with CMF [18]. Taken together, this data support that elderly TNBC patients in good general condition benefit from adjuvant chemotherapy and that less toxic regimens can be an efficient option.

Data in patients above 80 are scarce. In the English study, a minute proportion of patients > 80 years (1%) received chemotherapy [21] in agreement with the findings from the SEER database publications [17, 18]. We show that despite a median age of 81 years in our older group, the vast majority, almost 70%, died from their breast cancer and not from concomitant severe diseases. This is not true for BC overall where elderly patients often die from other diagnoses [23]. Since TNBC is an aggressive disease and the risk of recurrence is high during the first years after diagnosis, an older woman in a good performance status with TNBC often has a higher risk of dying from TNBC than from other causes.

Survival following distant metastasis was poor in our study for both age groups. As expected and potentially as a result of lack of primary treatment, the distant disease-free interval was shorter for elderly compared with younger patients with 13 and 24 months, respectively. Older patients had a poorer survival following distant metastasis, and this was significant when comparing patients over and under 75 years, concordant with previous published data including all breast cancer subtypes [24]. Taken into account that adjuvant chemotherapy was not given to a majority of the older, these patients may have had a less resistant disease at recurrence. However, our data show that the vast majority (90%) of the older patients did not even start any palliative chemotherapy. We cannot determine if this was due to a more advanced stage with a poor general condition, a result of delayed diagnosis or a general hesitation to initiate palliative chemotherapy from physicians or patients’ choice.

The metastatic patterns were in concordance with previous trials [8, 25]. We found no clear difference in metastatic spread other than the more frequently registered brain and liver metastases in the younger group. TNBC is a subgroup with a high risk of brain metastasis, in our whole population 40%, despite the commonly short post-recurrence survival. This is concordant with recently published data from a French multicenter database with more than 4000 BC patients with brain metastasis [26]. More than 50% of the younger patients were diagnosed with brain metastasis. The corresponding number among older was less than 20%, and the difference is probably partly a result of more frequent use of brain CT and MRI scans among younger patients. Moreover, older patients have a shorter survival time following recurrence, and thus death may occur before brain metastases have been developed.

We investigated if there was any biological difference between the age groups. Even though a majority of older patients had grade III tumors with a high proliferation rate, both grade and Ki67 were higher among young patients with TNBC. This is in concordance with a previous larger study comparing biology and survival in TNBC < 70 and ≥ 70 years [27]. The authors conclude that the equal survival found in this study, although no patient ≥ 70 years received adjuvant chemotherapy and 47% among the younger did, was explained by less aggressive tumors among old patients. The age comparison in our study is not identical, but we found a significant worse survival among TNBC > 75 compared with ≤ 75. This is probably explained by a higher proportion of adjuvant/neoadjuvant chemotherapy given among patients ≤ 75 years (92% among young and 11% among old patients) in our study, which is more recent. A previous study by Dreyer et al. [28] found a relation between age and histological subtype in patients with TNBC. Apocrine and lobular breast cancer seemed to be more common among old patients. However, subtypes other than ductal are rare and similarly we were in our study unable to find a statistical difference in histology in the different age groups.

Since a larger proportion of the young TNBC patients harbor a BRCA mutation, this could affect the prognosis and sensitivity to chemotherapy with more highly proliferative tumors in the younger group. The effect on BRCA mutation on outcome in TNBC is however debatable and a larger prospective trial showed that survival after 2 years is more favorable among BRCA mutation carriers with TNBC compared with wild-type TNBC but not at 5 years [29]. We do not consider it plausible that this affects our results in a significant way.

Our study has strength and weaknesses. Unlike register-based studies, in this up-to-date population-based cohort study with real-world data, we followed our patients for sufficient amount of time with medical record review and only 10 out of 524 patients were lost to follow-up. This provides strength to our results. To our very best knowledge, this is the first study that compared the biology, metastatic patterns and outcome of elderly versus younger patients with TNBC. The number of patients is small and this is especially obvious in the comparison between younger and older in the metastatic setting. Moreover, we lack information on BRCA status, further subgrouping of TNBC by use of gene expression profiling [30], as well as data on comorbidities and functional status.

In summary, older patients with TNBC in our study have a poor prognosis and as much as 68% of deaths in the older group were caused by TNBC. This can only be explained by the low proportion of patients in the older group that received adjuvant chemotherapy. Public health has improved in Sweden and life expectancy for women is now 84 years [31]. Chemotherapy should be considered in fit older patients with TNBC. However, there are still older patients with TNBC who are not suitable for adjuvant chemotherapy. General guidelines often do not take higher age into account. There may also be a hesitation from oncologists, patients and relatives when standard adjuvant chemotherapy is considered since older patients are more sensitive to adverse events [32]. This supports the use of the guidelines how to treat elderly with breast cancer [33] that includes geriatric assessment which we believe, at least in our region, is an underused but valuable tool.
